# Life Satisfaction and Work-Related Satisfaction among Anesthesiologists in Poland

**DOI:** 10.1155/2014/601865

**Published:** 2014-06-09

**Authors:** Ewelina Gaszynska, Michal Stankiewicz-Rudnicki, Franciszek Szatko, Andrzej Wieczorek, Tomasz Gaszynski

**Affiliations:** ^1^Department of Hygiene and Health Promotion, Medical University of Lodz, Poland; ^2^Department of Anesthesiology and Intensive Therapy, Medical University of Lodz, Poland; ^3^Department of Emergency Medicine and Disaster Medicine, Medical University of Lodz, Poland

## Abstract

The aim of the study was to assess the level of life and job satisfaction of Polish anesthesiologists and to explore the impact of extrinsic-hygiene and intrinsic-motivating determinants. * Materials and Methods.* A cross-sectional questionnaire study was conducted among consultant anesthesiologists in Lodz region. The questionnaire concerned patient care, burden, income, personal rewards, professional relations, job satisfaction in general, and life satisfaction. Respondents were asked to rate their level of satisfaction for each item on a seven-point Likert scale (1: extremely dissatisfied; 7: extremely satisfied). * Results.* 86.03% of anesthesiologists were satisfied with their economic status, 77.94% found their health status satisfactory, and 52.21% viewed their personal future optimistically. In general, 71.32% of anesthesiologists were satisfied with their current job situation. Among the less satisfying job aspects were work-related stress (2.49; SD = 1.23), administrative burden (2.85; SD = 1.47), workload (3.63; SD = 1.56), and leisure time (3.09; SD = 1.44). * Conclusions.* Considerable work-related stress leads to job dissatisfaction among anesthesiologists. There is an association between job satisfaction and health status, social life, and economic status. Working for long hours by anesthesiologists results in a high risk of burnout.

## 1. Background

Job satisfaction is defined as positive feelings of individuals towards their job. According to the Two-Factor Theory of job satisfaction laid out by Herzberg and colleagues, the factors influencing satisfaction levels can be divided into intrinsic-motivating factors (recognition, work tasks, and responsibility) and extrinsic-hygiene factors (job security, working conditions, and salary) [1]. The presence of intrinsic motivators increases satisfaction levels, whereas the lack of extrinsic-hygiene factors may cause dissatisfaction. These factors acting on one another have been shown to be an important modifier of the quality of medical care. Highly satisfied workers care about the quality of their work, are more productive, and feel responsible for the working environment. Staff satisfaction should be a strategic aim of any healthcare system as it facilitates its organization and contributes to cost reduction. Different studies reveal that doctors, anesthesiologists in particular, suffer from occupational stress and burnout [2].

Over the last years working conditions of anesthesiologists in Poland have changed considerably and new forms of employment have emerged. Due to limited healthcare funding, hospitals have faced meticulous financial scrutiny and pressure to generate profits. This entailed a necessity to reduce the costs associated with the services provided (using cheapest treatment options that are not equally effective as other available ones) along with the need to improve workforce productivity. Administrative workload has increased as well. The media-created image of doctors was instrumental in the deterioration of social recognition and respect for medical profession. Doctors have to deal with the threat of malpractice litigation more than ever. On the other hand, maximum working hours have been reduced and, as a result of the continuous educational obligation, employers are now required to grant paid time-off to employees for further education and professional skills development. At a time of healthcare reorganization in Poland all of the mentioned factors may influence job satisfaction. Research on job satisfaction of anaesthesia providers was carried out in many countries. In Poland still little is known about the work-related well-being of anesthesiologists and the factors that could improve their situation.

The aim of this study was to assess the level of life and job satisfaction of Polish senior specialist anesthesiologists and to explore the impact of extrinsic-hygiene and intrinsic-motivating determinants. A potential modification of these factors may lead to the improvement of anaesthesiologists' performance in the working environment.

## 2. Materials and Methods

The study was approved by the Medical University of Lodz Research Ethics Board (number RNN/134/13/KB Lodz, Poland). In January 2013 postal survey with a covering letter was sent to all senior specialist anesthesiologists working within Lodzkie Voivodeship. The participants' names came from the register of Voivodeship Consultant updated in the year 2012, which covers all anesthesiologists employed in the Voivodeship. A cross-sectional questionnaire study was conducted among 177 senior specialist anesthesiologists from 14 hospitals in the general area of Lodz. Participants were asked to complete a questionnaire, which was a modified version of the questionnaire introduced by Bovier et al. and was returned in a prepaid envelope by mail. It included questions about each single intrinsic and extrinsic item of job satisfaction: patient care, burden, income, personal rewards, professional relations, and job satisfaction in general [3, 4]. Other variables that are known to influence job satisfaction level were also taken into account: sociodemographic data—age, gender, marital status, years since graduation, mean number of working hours a week, type of employment, and number of employers (number of employment places)—and life satisfaction—standard of living, health status, predictions for the future, and social and family life. Respondents were asked to rate their level of satisfaction with each item on a seven-point Likert scale (1: extremely dissatisfied; 7: extremely satisfied).

### 2.1. Statistical Analysis

Data were summarized using means and standard deviations and were presented as frequency counts and percentages. Pearson's *r* correlation coefficients were computed between separate aspects of life and job satisfaction in general. Gender differences in job satisfaction and differences between burnout and satisfied groups were compared using the nonparametric, Mann-Whitney *U* test. An explanatory factor analysis, principal component analysis with varimax rotation, was conducted in order to define meaningful constituents (dimensions) in terms of work satisfaction among anesthesiologists. Hence, a five-dimensional model was used: patient care (autonomy in treating patients; quality of care one can provide), burden (workload; time available for family, friends, or leisure; work-related stress; administrative burden), income-prestige (current income; social status and respect), personal rewards (intellectual stimulation; opportunity for continuing medical education; enjoyment of work), and professional relations (with peers, nurses, and other staff). Internal consistency (Cronbach's *α*) of the analyzed subscales is as follows: patient care: *α* = 0.78, burden: *α* = 0.70, income-prestige: *α* = 0.55, personal rewards: *α* = 0.81, and professional relations: *α* = 0.71.

## 3. Results 

The response rate was 76.84% (136/177); 47.79% (65) were men. Average age of the participants was 48.74 years (SD = 9.96; quartiles: 41, 49, and 56.50). Private hospitals were not main employers for any of the respondents. Half of the anesthesiologists were employed in one hospital, 27.94% were employed in 2, and 22.06% were employed in 3 or more. Most anesthesiologists worked under a fixed-term or permanent employment contract and reconciled this with self-employment, that is, contract for services. On average 22.93 years (SD = 10.34; quartiles: 15, 23.5, and 31 respondents graduated from medical school ago) passed since the respondents graduated from medical school.

### 3.1. Life Satisfaction

Most participants were content (extremely satisfied, satisfied, or rather satisfied) with different aspects of their life: economic status, health status, outlooks for the future, and social and family life—86.03%, 77.94%, 52.21%, 55.15%, and 74.99%, respectively.

#### 3.1.1. Job Satisfaction in General

In general, 71.32% of anesthesiologists were satisfied with their current job situation; most, however, (62.65%) used the term “rather satisfied.” Respondents declaring general satisfaction with their job were significantly more content with its different aspects, patient care (*P* < 0.001), income-prestige (*P* < 0.001), personal rewards (*P* < 0.001), burden (*P* = 0.009), professional relations (*P* = 0.024), and life in general, than the dissatisfied ones (Table 1). Family life was the only aspect that these differences did not apply to. The satisfied participants rated their health and economic status significantly higher (*P* < 0.001). They also viewed their future more optimistically (*P* < 0.001) and were more satisfied with their social (*P* = 0.036) and family life (*P* = 0.007).

Table 2 presents statistics describing satisfaction with 13 different job aspects and current job situation. Anesthesiologists' contentment with different job aspects was diverse. Best results were obtained for professional relations with nurses and other staff (5.40; SD = 0.98) and relations with other doctors (5.10; SD = 1.23) as well as enjoyment of work (5.21; SD = 1.14). Among the less satisfying job aspects were factors connected with work-related stress (2.49; SD = 1.23), administrative burden (2.85; SD = 1.47), workload (3.63; SD = 1.56), and leisure time (3.09; SD = 1.44).

### 3.2. Patient Care

61.8% of the examined anesthesiologists were satisfied with their autonomy in treating patients. 64.6% were satisfied with the quality of care they can provide. A significant relation was observed between the autonomy in patient treatment and satisfaction with the quality of provided care.

### 3.3. Burden

The lowest satisfaction scores were found for burden. Only 36% were satisfied with their workload while 54.41% were dissatisfied. 72.06% found the time they can devote to their friends and family or leisure activities insufficient. 77.32% were dissatisfied with work-related stress (assessed as too high). 69.12% were dissatisfied with administrative burden.

### 3.4. Income-Prestige

More than a half of participating anesthesiologists (55.3%) expressed satisfaction with their current income. One in every two were satisfied with their social status and the respect they have.

### 3.5. Personal Benefits

Most anesthesiologists (80.34%) declare they enjoy their work. Around a half believes that the prospects for further education and professional development are good. 47.06% were satisfied with intellectual stimulation they gain at work.

### 3.6. Professional Relations

78.78% were satisfied with the relations with their peers and 86.02% with the relations with nurses and other staff.

### 3.7. Relation to Sociodemographics and Life Satisfaction

Job satisfaction in general had strongest correlation with health status and predicted future followed by social life and economic status. Statistically significant (*P* < 0.001) correlation was found between satisfaction with social life (*r* = 0.40), health status (*r* = 0.30), and burnout, with time available for friends and family being the most important factor. Outlooks for the future (predicted future) were significantly (*P* < 0.001) influenced by health status, income, intellectual stimulation at work, and work enjoyment.

In our analysis, working hours were associated with the perceived burden level. Burnout level was significantly higher in anesthesiologists who worked more than 60 hours a week (Table 3). They assessed their health status and social and family life (*P* ≤ 0.05) as significantly worse than the physicians who work shorter (Table 4). Those working more than 20 years gave a less favourable assessment of their career outlooks (predicted future) and family and social life (*P* ≤ 0.05).

No statistically significant differences were found between men and women in the examined group. Different aspects of job satisfaction were also not influenced by marital status, place of residence, years since graduation, and number of jobs (employment places).

## 4. Discussion

The overall job satisfaction level among anesthesiologists in our study is comparable to the findings of other studies in different countries and is estimated at around 71–75% [5–7]. Our results demonstrate that anesthesiologists working longer hours are more prone to burnout and are more likely to be dissatisfied with different aspects of life (except family life). This trend is progressive with age.

Some studies point to more demanding family life responsibilities and discrimination in the work environment as causes for a lower satisfaction level and greater work-related stress issues among women. Others, including ours, cannot confirm these findings on the basis of observed survey results [8, 9]. No differences in any of the examined aspects of job satisfaction between participants with various length of service have been recorded, which were observed elsewhere in Europe [5, 10, 11].

The main positive determinants of job satisfaction among Polish anesthesiologists, similar to their Finnish and Swiss counterparts, were the quality of care one can provide and autonomy in patient treatment [5, 11]. Most anesthesiologists are content with their income levels. This is, however, at the expense of long working hours, often exceeding the EU norms. According to a report by the Ministry of Health, based on questionnaires obtained from 384 hospitals, the average monthly gross income of a senior specialist anesthetist in mid-2008 was 7211 PLN. Basic salary usually constitutes a half of the total income, the rest being earnings for staying on duty. Self-employed doctors earn up to 300% more. They often stay on duty eight days a month, with record breakers even as many as 20. This is where the earnings of more than 15000 PLN, declared by half of them, come from. More than 83% of doctors work longer than allowed by the EU standards [12]. One in ten doctors works continuously for more than 13 hours a day (also on weekends) and one in twenty even as long as 18 hours. Often doctors work continuously for more than 30 hours (staying on duty for 24 hours and then the standard 8-hour working day), especially in smaller, understaffed hospitals [13]. Anesthesiologists usually earn more but work longer as well. In 2011 the basic salary among senior anesthesiologists, without any extra income for staying on duty, was between 2499 PLN and 11500 PLN, median: 4080 PLN (83% of respondents). Self-employed anesthesiologists earned between 26 PLN/h and 120 PLN/h in public hospitals, median: 67 PLN/h, and between 45 PLN/h and 200 PLN/h in private hospitals, median: 94,5 PLN/h. Anesthesiologists who completed the questionnaire in the ministry's study declared they had to work continuously for 24 hours 6 times a month on average; their mean working time was 267 hours a month, which is in line with the data on anesthesiologists' working time we obtained in our study [14].

Anesthesiologists are exposed to greater levels of stress than normative groups and as a result more frequently engage in alcohol and drug abuse, suffer from mental disorders, and find it more difficult to reconcile work with family life [15–18].

In Poland and Germany anesthesiologists experience more stress and are at a greater risk of burnout than general practitioners [3, 19]. Stress is caused by the responsibility for providing safe and high quality medical services, dealing with challenging medical situations, and making ethically and therapeutically difficult decisions, which anesthesiologists face every day. Working in the operating theatre, the ICU, making preoperative assessment, treating chronic and acute pain, or working in an emergency department anesthesiologists provide services for as many as 50–60% of hospitalized patients [20, 21]. That is why the proportion of the amount of workload, both with patients and with administrative burden, to leisure time remains unsatisfactory. Although data differs by country, it is estimated that as many as 25% of anesthesiologists are in the burnout high risk group [11, 22–24]. Authors of the study conducted among perioperative clinicians in the United States reported higher burnout scores in physicians than nurse anesthetists and the highest ones among residents [9]. Young anesthesiologists during their training are more prone to burnout and depression compared to people of similar age but different specialization. Surveys conducted in Turkey and the United States also revealed that anesthesiologists, especially without support from the family, are more likely to report suicidal thoughts [18, 25].

Good peer relations have been proven to reduce probability of burnout. Although most participants of our study described their relations with other medical staff as positive, anesthesiologists often have to deal with a lack of positive feedback from patients and colleagues. This can be attributed to the fact that anesthesiologists in the operating theatre are often perceived as mere comfort providers for surgeons, rather than critical elements of the process they are in practice.

Anesthesiologists evaluated their overall situation at work more favourably than primary care physicians (4.72) in Poland. Participants of our study, however, viewed their social status as lower and believed they received less respect from other people than general practitioners. Indeed, research carried out among patients and their relatives in Polish hospitals reveals that more than 2/3 of patients do not know what the work of an anesthesiologist involves and that anesthesiologists are doctors [26]. Despite the threat of stress and burnout most anesthesiologists are more satisfied with their jobs than GPs.

High job satisfaction levels among doctors reduce their susceptibility to burnout and mental disorders. Dissatisfied medical staff are more prone to burnout which decreases patient safety. A satisfied doctor is more committed to work and more willing to make sacrifices, exhibits greater productivity levels and lowers labor costs, contributes to patient satisfaction, and is a prerequisite for a good work environment [27].

## 5. Conclusions

1. Job dissatisfaction among anesthesiologists is caused by heavy stress exposure. Work enjoyment is a protective factor against dissatisfaction.
2. Anesthesiologists are at a high risk for burnout, especially when working long hours.
3. The situation of anesthesiologists in Poland could be improved by enforcing shorter working hours and lower stress exposure through the introduction of standards for medical practice.

## Figures and Tables

**Table 1:** Computed statistical parameters (mean, SD, and quartiles) for each averaged scoring referring to patient care, burden, income-prestige, personal rewards, and professional relations and different aspects of life among surveyed anesthesiologists being generally satisfied versus dissatisfied^a, b, c^.

Investigated “dimensions” of work satisfaction or aspect of life	Job satisfaction in general—declared general satisfaction of work			Job satisfaction in general—declared general dissatisfaction of work^b^			*P* value^c^
	Mean	SD	Quartiles	Mean	SD	Quartiles	
Patient care	4.78	1.10	4; 5; 5.5	3.69	1.27	2.5; 4; 5	*P* < 0.001
Burden	3.17	1.05	2.25; 3; 4	2.63	0.92	2; 2.5; 3.25	*P* = 0.009
Income-prestige	4.56	0.94	4; 4.5; 5.5	3.69	1.12	3; 3.5; 4.5	*P* < 0.001
Personal rewards	4.83	0.98	4.33; 5; 5.67	3.97	1.19	3; 4; 5	*P* < 0.001
Professional relations	5.40	0.82	5; 5.5; 6	4.86	1.21	4; 5; 6	*P* = 0.024
Material status	5.48	0.86	5; 6; 6	4.79	1.22	4; 5; 6	*P* < 0.001
Health status	5.41	0.94	5; 5; 6	4.49	1.30	4; 5; 5	*P* < 0.001
Predicted future	4.77	1.04	4; 5; 6	4.00	1.08	4; 4; 5	*P* < 0.001
Social life	4.60	1.46	4; 5; 6	3.87	1.94	2; 4; 5	*P* = 0.036
Family life	5.34	1.40	5; 6; 6	5.00	1.59	4; 5; 6	(NS^d^)

^a^1: extremely dissatisfied; 2: dissatisfied; 3: rather dissatisfied; 4: neither dissatisfied nor satisfied; 5: rather satisfied; 6: satisfied; 7: extremely satisfied.

^b^“Satisfied anesthesiologists” indicated the score ranging from 5 to 7; “dissatisfied anesthesiologists” chose the score ranging from 1 to 4. The Mann-Whitney *U* test was carried out.

**Table:** (a)

Elements to be assessed	Statistical parameters			Computed frequencies for each scoring^a^
	Mean	SD	Quartiles	
Job situation in general	4.86	1.18	4; 5; 6	2 persons (1.47%) 3 persons (2.21%) 13 persons (9.56%) 21 persons (15.44%) 58 persons (42.65%) 33 persons (24.26%) 6 persons (4.41%)

Patient care	4.46	1.25		

Autonomy in treating patients	4.43	1.45	3; 5; 5	4 persons (2.94%) 11 persons (8.09%) 27 persons (19.85%) 10 persons (7.35%) 53 persons (38.97%) 26 persons (19.12%) 5 persons (3.68%)

Quality of care one can provide	4.50	1.31	3; 5; 5	2 persons (1.47%) 9 persons (6.62%) 27 persons (19.85%) 10 persons (7.35%) 58 persons (42.65%) 29 persons (21.32%) 1 person (0.74%)

Burden	3.01	1.04		

Workload	3.63	1.56	3; 3; 5	13 persons (9.56%) 19 persons (13.97%) 42 persons (30.88%) 13 persons (9.56%) 33 persons (24.26%) 13 persons (9.56%) 3 persons (2.21%)

Time available for family, friends, or leisure	3.09	1.44	2; 3; 4	16 persons (11.76%) 35 persons (25.74%) 47 persons (34.56%) 6 persons (4.41%) 24 persons (17.65%) 7 persons (5.14%) 1 person (0.74%)

Work-related stress	2.49	1.23	1; 2; 3	38 persons (27.94%) 31 persons (22.79%) 36 persons (26.47%) 26 persons (19.12%) 3 persons (2.21%) 2 persons (1.47%) None (0.00%)

Administrative burden	2.85	1.47	2; 3; 4	27 persons (19.85%) 38 persons (27.94%) 29 persons (21.33%) 20 persons (14.71%) 15 persons (11.03%) 6 persons (4.41%) 1 person (0.74%)

^a^1: extremely dissatisfied; 2: dissatisfied; 3: rather dissatisfied; 4: neither dissatisfied nor satisfied; 5: rather satisfied; 6: satisfied; 7: extremely satisfied.

**Table:** (b)

Elements to be assessed	Statistical parameters			Computed frequencies for each scoring^a^
	Mean	SD	Quartiles	
Income-prestige	4.31	1.06		

Current income	4.20	1.38	3; 5; 5	5 persons (3.68%) 12 persons (8.82%) 30 persons (22.06%) 14 persons (10.29%) 55 persons (40.44%) 19 persons (13.97%) 1 person (0.74%)

Social status and respect	4.43	1.18	4; 4.5; 5	None (0.00%) 7 persons (5.15%) 25 persons (18.38%) 36 persons (26.47%) 41 persons (30.15%) 25 persons (18.38%) 2 persons (1.47%)

Personal rewards	4.59	1.11		

Intellectual stimulation	4.25	1.37	3; 4; 5	3 persons (2.21%) 15 persons (11.03%) 20 persons (14.70%) 34 persons (25.00%) 37 persons (27.21%) 25 persons (18.38%) 2 persons (1.47%)

Opportunity for continuing medical education	4.29	1.41	3; 5; 5	1 person (0.74%) 16 persons (11.76%) 30 persons (22.06%) 17 persons (12.50%) 42 persons (30.88%) 27 persons (19.85%) 3 persons (2.21%)

Enjoyment of work	5.21	1.14	5; 5; 6	1 person (0.74%) 3 persons (2.21%) 7 persons (5.15%) 16 persons (11.76%) 48 persons (35.29%) 50 persons (36.76%) 11 persons (8.09%)

Professional relations	5.25	0.98		

Relations with peers	5.10	1.23	5; 5; 6	2 persons (1.47%) 5 persons (3.68%) 9 persons (6.61%) 13 persons (9.56%) 47 persons (34.56%) 53 persons (38.97%) 7 persons (5.15%)

Relations with nurses and other staff	5.40	0.98	5; 6; 6	1 person (0.74%) None (0.00%) 5 persons (3.68%) 13 persons (9.56%) 46 persons (33.82%) 61 persons (44.85%) 10 persons (7.35%)

^a^1: extremely dissatisfied; 2: dissatisfied; 3: rather dissatisfied; 4: neither dissatisfied nor satisfied; 5: rather satisfied; 6: satisfied; 7: extremely satisfied.

**Table 3:** Sociodemographic discrete predictors (along with corresponding *P* values) of each scoring referring to job satisfaction in general, patient care, burden, income-prestige, personal rewards, and professional relations among surveyed anesthesiologists.

Sociodemographic variables/predictors^a^	Surveyed “dimensions” of work satisfaction					
	Job satisfaction in general	Patient care	Burden	Income-prestige	Personal rewards	Professional relations
Gender (i) Male (ii) Female	(NS^b^) 4.89 4.83	(NS^b^) 4.44 4.49	(NS^b^) 3.11 2.93	(NS^b^) 4.20 4.42	(NS^b^) 4.56 4.61	(NS^b^) 5.25 5.24
Marital status (i) Single (ii) Married	(NS^b^) 4.89 4.85	(NS^b^) 4.51 4.45	(NS^b^) 2.89 3.06	(NS^b^) 4.19 4.36	(NS^b^) 4.44 4.64	(NS^b^) 5.29 5.23
Place of living (i) Big city (>20 000 citizens) (ii) Small town (<20 000 citizens) (iii) Rural area	(NS^b^) 4.84 4.80 5.20	(NS^b^) 4.38 4.64 4.13	(NS^b^) 2.94 3.11 2.95	(NS^b^) 4.13 4.43 4.60	(NS^b^) 4.48 4.73 4.44	(NS^b^) 5.18 5.23 5.60
Years since graduation (i) ≤10 (ii) 11–20 (iii) 21–30 (iv) >30	(NS^b^) 5.31 5.04 4.57 4.83	(NS^b^) 4.90 4.24 4.23 4.64	(NS^b^) 3.44 3.14 2.69 3.08	(NS^b^) 4.37 4.37 4.05 4.61	(NS^b^) 5.09 4.39 4.47 4.51	(NS^b^) 5.63 5.39 4.90 5.36
Working hours (i) ≤60 (ii) >60	(NS^b^) 4.95 4.73	(NS^b^) 4.54 4.37	(*P* < 0.001) 3.30 2.61	(NS^b^) 4.39 4.20	(NS^b^) 4.63 4.52	(NS^b^) 5.31 5.16
Number of employment places (i) One (ii) Two or more	(NS^b^) 4.85 4.87	(NS^b^) 4.49 4.44	(NS^b^) 3.11 2.92	(NS^b^) 4.29 4.34	(NS^b^) 4.50 4.67	(NS^b^) 5.13 5.37

^a^One-way ANOVA or test for trend across ordered groups has been carried out when appropriate.

^b^NS: (statistically) not significant.

**Table 4:** Sociodemographic discrete predictors (along with corresponding *P* values) of analyzed aspects of life in surveyed anesthesiologists.

Sociodemographic variables/predictors^a^	Aspects of life				
	Material status	Health status	Predicted future	Social life	Family life
Gender (i) Male (ii) Female	(NS^b^) 5.25 5.32	(NS^b^) 5.32 5.07	(NS^b^) 4.66 4.45	(NS^b^) 4.66 4.14	(NS^b^) 5.45 5.06
Marital status (i) Single (ii) Married	(NS^b^) 5.20 5.32	(NS^b^) 5.14 5.15	(NS^b^) 4.51 4.56	(NS^b^) 4.43 4.45	(*P* = 0.001) 4.57 5.48
Place of living (i) Big city (>20 000 citizens) (ii) Small town (<20 000 citizens) (iii) Rural area	(NS^b^) 5.13 5.32 5.80	(NS^b^) 5.15 5.20 4.93	(NS^b^) 4.58 4.58 4.33	(NS^b^) 4.42 4.36 4.40	(NS^b^) 5.10 5.44 5.07
Years since graduation (i) ≤10 (ii) 11–20 (iii) 21–30 (iv) >30	(NS^b^) 5.46 5.39 5.12 5.33	(*P* = 0.011) 5.69 5.30 4.80 4.76	(*P* < 0.001) 5.31 4.70 4.24 4.36	(*P* = 0.002) 5.38 4.48 4.02 4.14	(*P* = 0.028) 5.77 5.61 4.80 5.25
Working hours (i) ≤60 (ii) >60	(NS^b^) 5.39 5.14	(*P* = 0.025) 5.31 4.91	(NS^b^) 4.60 4.48	(*P* = 0.008) 4.74 3.89	(*P* = 0.015) 5.50 4.88
Number of employment places (i) One (ii) Two or more	(NS^b^) 5.28 5.29	(NS^b^) 5.06 5.24	(NS^b^) 4.43 4.68	(NS^b^) 4.40 4.38	(NS^b^) 5.38 5.10

^a^One-way ANOVA or test for trend across ordered groups has been carried out when appropriate.

^b^NS: (statistically) not significant.
